# Analytical Validation of GFR_NMR_: A Blood-Based Multiple Biomarker Assay for Accurate Estimation of Glomerular Filtration Rate

**DOI:** 10.3390/diagnostics12051120

**Published:** 2022-04-30

**Authors:** Markus Fuhrmann, Amauri Schwaeble Santamaria, Renee Scott, Jeffrey W. Meeusen, Marianna Fernandes, John Venz, Victoria Rothe, Frank Stämmler, Jochen Ehrich, Eric Schiffer

**Affiliations:** 1 Department of Research and Development, numares AG, 93053 Regensburg, Germany; markus.fuhrmann@numares.com (M.F.); amauri.schwaeblesantamaria@numares.com (A.S.S.); john.venz@numares.com (J.V.); victoria.rothe@numares.com (V.R.); frank.staemmler@numares.com (F.S.); 2 Department of Laboratory Medicine and Pathology, Mayo Clinic, Rochester, MN 55905, USA; scott.renee@mayo.edu (R.S.); meeusen.jeffrey@mayo.edu (J.W.M.); 3 Boston Heart Diagnostics, Framingham, MA 01702, USA; mfernandes@bostonheartdx.com; 4 Children’s Hospital, Hannover Medical School, 30625 Hannover, Germany; ehrich.jochen@mh-hannover.de

**Keywords:** glomerular filtration rate, eGFR, metabolite, NMR, analytical validation, linearity, precision, trueness, interference, stability

## Abstract

Accurate and precise monitoring of kidney function is critical for a timely and reliable diagnosis of chronic kidney disease (CKD). The determination of kidney function usually involves the estimation of the glomerular filtration rate (eGFR). We recently reported the clinical performance of a new eGFR equation (GFR_NMR_) based on the nuclear magnetic resonance (NMR) measurement of serum myo-inositol, valine, and creatinine, in addition to the immunoturbidometric quantification of serum cystatin C, age and sex. We now describe the analytical performance evaluation of GFR_NMR_ according to the Clinical and Laboratory Standards Institute guidelines. Within-laboratory coefficients of variation (CV%) of the GFR_NMR_ equation did not exceed 4.3%, with a maximum CV% for repeatability of 3.7%. Between-site reproducibility (three sites) demonstrated a maximum CV% of 5.9%. GFR_NMR_ stability was demonstrated for sera stored for up to 8 days at 2–10°C and for NMR samples stored for up to 10 days in the NMR device at 6 ± 2°C. Substance interference was limited to 4/40 (10.0%) of the investigated substances, resulting in an underestimated GFR_NMR_ (for glucose and metformin) or a loss of results (for naproxen and ribavirin) for concentrations twice as high as usual clinical doses. The analytical performances of GFR_NMR,_ combined with its previously reported clinical performance, support the potential integration of this NMR method into clinical practice.

## 1. Introduction

Chronic kidney disease (CKD) is a leading public health burden affecting more than 50 million people worldwide [1,2,3]. Kidney Disease: Improving Global Outcomes (KDIGO) defines CKD as abnormalities of kidney structure or function, present for >3 months, with negative health implications. CKD criteria include a decreased glomerular filtration rate (GFR < 60 mL/min/1.73 m^2^) and/or 1 or more markers of kidney damage, such as albuminuria [4,5].

A Precise determination of GFR requires measurement of an exogenous tracer substance that is both freely filtered by the kidney and does not undergo metabolism, tubular secretion or absorption. As such, the gold standard for determining GFR is inulin clearance [2,6,7,8,9]. However, the use of such exogenous markers is impractical and costly. Therefore, endogenous GFR markers, such as serum creatinine and/or cystatin C, are measured to estimate GFR using validated eGFR equations in routine clinical practice [9,10,11,12,13,14,15].

Although in use for decades, eGFR equations present important limitations. These include the impact of body mass, diet, and age, which can influence metabolite production, tubular secretion, reabsorption, or extra-renal excretion [3,10,13,16,17]. Such limiting factors may result in significant deviations of eGFR from tracer-measured GFR (mGFR), incorrect CKD staging, and different rates of GFR decline [3,8,10,13,16,17], all of which limit the clinical utility of proposed eGFR equations. 

In addition to biological limitations affecting clinical performance of eGFR, the analytical performance of metabolite measurements by a particular method can greatly impact the precision and accuracy of eGFR equations [8,18,19,20,21]. The factors potentially influencing the analytical performance of an eGFR assay include analytical precision and linearity of the measurement method, the impact of substances interfering with marker quantification in the sample, and sample stability [8,10,19,20,22,23,24,25]. Assay calibration has allowed for the reduction in errors associated with metabolite measurement, as well as reduce inter-laboratory variability of eGFR methods [3,18,21,26]. Altogether, analytical validation is essential for the standardization and implementation of new eGFR assays in clinical laboratories.

We have recently reported the clinical performance of a new blood-based assay, GFR_NMR_, for the accurate estimation of GFR (normalized to 1.73 m^2^ body surface area) in subjects with and without CKD [15]. The GFR_NMR_ equation is based on measurements of serum myo-inositol, valine, creatinine, and cystatin C, and integrates age and sex [15]. Practically, the GFR_NMR_ assay includes the simultaneous measurement of serum creatinine, valine and myo-inositol by nuclear magnetic resonance (NMR) spectroscopy, in addition to serum cystatin C measurement using a validated particle-enhanced turbidimetric immunoassay (PETIA) [27,28]. Clinical performance validation demonstrated a lower bias of the GFR_NMR_ equation to mGFR and a higher P15 accuracy compared to recommended eGFR equations (including Chronic Kidney Disease Epidemiology Collaboration [CKD-EPI] and European Kidney Function Consortium [EKFC] equations) [15].

We now describe the analytical performance validation of the GFR_NMR_ assay, including both the analytical validation of the NMR measurements of serum creatinine, valine, and myo-inositol, and of the resulting GFR_NMR_ equation. We assessed metabolite measurements by NMR and GFR_NMR_ scores for repeatability and reproducibility, range of linearity, result stability in standard laboratory conditions, and the influence of potentially interfering substances. We show that metabolite measurement by NMR and GFR_NMR_ scores present analytical performances compatible with routine clinical practice, with high repeatability and reproducibility, a broad range of linearity, result stability, and limited influence of potentially interfering substances. 

## 2. Materials and Methods

### 2.1. Blood Serum

Human serum was obtained from the Blood Donation Service of the Bavarian Red Cross as single-donor material prepared from 0.5 L of whole blood. The study used anonymized data (no personal reference can be established) and consequently was not subject to ethical consultation by the responsible ethics committee of the Bavarian Medical Association. All donating subjects gave written informed consent to use residual serum for research according to the Declaration of Helsinki (revised version of the 64th WMA General Assembly, Fortaleza, Brazil, October 2013), and following the standards of ICH-GCP E6 (R2).

### 2.2. Serum Cystatin C Measurements

Serum cystatin C measurements were performed with the Tina-Quant Cystatin C Gen.2 assay (Roche) on cobas 8000 Modular Analyzer (Roche) by an external accredited laboratory (Labor Staber, Munich, Germany), using the human serum certified reference material (CRM) ERM-DA471/IFCC (European Commission Joint Research Centre, Institute for Reference Materials and Measurements, Belgium) as cystatin C calibrator [29]. 

### 2.3. NMR Sample Preparation 

Samples were prepared by mixing 540 µL serum with 60 µL of AXINON serum additives solution 2.0. A total volume of 600 µL was filled into a 5 mm NMR tube and capped with a barcoded cap for identification. Calibrator and quality control samples were prepared by filling 600 µL of AXINON serum calibrator or control 2.0, respectively, into a 5 mm NMR tube and capping with a barcoded cap for identification.

### 2.4. NMR Analysis and Biomarker Quantification

NMR analysis was performed as described elsewhere [30]. Prepared samples were pre-heated at 37 °C for 7.5 min before NMR measurement in a Bruker Avance III 600 MHz and a 5 mm PATXI probe equipped with automatic Z gradients shimming. A modified version of the CPMG pulse sequence was used. ^1^H-NMR spectra were recorded using a spectral width of 20 ppm, with a recycling delay of 1.5 s, 16 scans, and a fixed receiver gain of 50.4. A cycling time d2 of 8 ms was used together with a corresponding T2 filter of 112 ms. Mixing time τ between 2 consecutive spin echoes was 400 µs. NMR data were automatically phase- and baseline-corrected using the lactate doublet at 1.32 ppm as reference. Metabolite quantification used curve-fitted pseudo-Voigt profiles [30]. In case of severe or frequent overlapping signals derived from interfering substances in the targeted spectral region, simultaneous fitting for the metabolite and the interfering substance(s) were performed [31].

### 2.5. Detection Capability (LoB, LoD, LoQ) 

The determination of limit of blank (LoB), limit of detection (LoD), and limit of quantification (LoQ) for creatinine, valine and myo-inositol NMR measurements was performed according to the Clinical and Laboratory Standards Institute (CLSI) guideline EP17-A2 [32]. To generate blank fractions for LoB, aliquots of 4 different serum samples were cleared of small metabolites by dialysis against 1x phosphate buffered saline (PBS) supplemented with 10 mg/dL sodium-(L) Lactate, using Slida-A-Lyzer Mini Dialysis Devices with a molecular weight cutoff of 20,000 kDa (ThermoFisher Scientific, Waltham, MA, USA). A compilation of measurements from a number of samples, rather than a single sample, was generated as recommended [32]. For the determination of LoB, a total of 45 replicates for each of the 4 different dialyzed serum pools were measured within three days using three batches of reagents. The required low-level samples for LoD and LoQ determination were prepared by mixing normal serum samples with dialyzed serum samples in different levels until the desired concentration ranges were obtained. For the determination of LoD and LoQ, a total of 45 replicates for each of the different mixed low-level serum sample pools were measured within 3 days using three batches of reagents. 

### 2.6. Linearity 

Linearity of creatinine, valine and myo-inositol NMR measurements were determined according to CLSI guideline EP6-A [33]. To generate a low-level fraction, an aliquot of this serum was cleared of small metabolites by dialysis against 1× PBS supplemented with 10 mg/dL of sodium-(L) Lactate as described above. To generate a very high-level fraction, another aliquot of the serum was supplemented with creatinine (Sigma Aldrich, St. Louis, MO, USA), valine (Sigma Aldrich) and myo-inositol (Sigma Aldrich) to final concentrations >1 mmol/L for creatinine and valine, and >0.4 mmol/L for myo-inositol. A total of 11 equidistant concentration levels were prepared by linear intermixture of the high- and low-level fractions ranging from 100% high level to 0% low level, as recommended [33]. 

### 2.7. Precision

#### 2.7.1. Single-Site Precision

Single-site precision was determined for the creatinine, valine, and myo-inositol analyte measurements and for the GFR_NMR_ equation. Repeatability, between-run precision, between-day precision and within-laboratory precision were calculated according to CLSI guideline EP05-A3 [34]. Human serum from nine (analyte measurement precision) or 4 (GFR_NMR_ precision) donors was prepared in aliquots of 1.8 mL for a total of 20 experimental days and 2 runs per day. Aliquots were stored at −80 °C until use. The analyte measurement repeatability study was conducted over 20 days with 2 runs per day and 3 replicates per sample, using 9 serum sample batches chosen to cover low to high analyte concentrations (1080 measurements). The GFR_NMR_ repeatability study was conducted over 20 days with 2 runs per day and three replicates per sample, using 4 serum sample batches chosen to cover GFR_NMR_ values from impaired (below 60 mL/min/1.73 m^2^) to physiological GFR around 90 mL/min/1.73 m^2^ (480 measurements). 

#### 2.7.2. Multi-Site Precision 

Multi-site precision was determined for the GFR_NMR_ equation. Repeatability, between-day precision, within-laboratory precision, and reproducibility were calculated according to CLSI guideline EP05-A3 [34]. Human serum from 4 donors was prepared in aliquots of 4.5 mL for 5 experimental days and one run per day on three different devices. Aliquots were stored at −80 °C until use. The reproducibility study was conducted on 3 devices at different laboratory sites, over 5 days, with 1 run per day, and 6 replicates per sample using 4 serum sample batches chosen to have GFR_NMR_ values covering the range from below 60 up to 90 mL/min/1.73 m^2^ (360 measurements). 

### 2.8. Method Comparison (Trueness) 

Trueness of creatinine, valine and myo-inositol analyte measurements was determined using a reference method, when possible, or reference materials with known concentrations, according to the CLSI guidelines EP09-A3 and EP15-A3 [35,36].

#### 2.8.1. Creatinine

A total of 150 serum samples were prepared as aliquots and a subset of 50 samples were analyzed at 3 independent sites. Additionally, creatinine reference measurements were performed with the Creatinine plus ver.2 (CREP2) enzymatic assay (Roche) on the legally marketed device cobas 8000 Modular Analyzer (Roche) by an external accredited laboratory (Labor Staber, Munich, Germany).

#### 2.8.2. Valine

A total of 120 pooled human serum samples (4 mL each) were prepared from 2 to 3 single donor sera. A first set of 100 samples was measured without any modification. A second set of 10 samples was spiked with valine covering the high range of concentrations >1 mmol/L. A third set of 10 samples was dialyzed against 1× PBS supplemented with ≥10 mg/dL lactate, using Slida-A-Lyzer Mini Dialysis Devices with a molecular weight cutoff of 10,000 kDa (ThermoFisher Scientific). These samples were subsequently spiked with valine covering the lower concentration range from 0.2 to 0.4 mmol/L. Valine reference measurements were performed using the *AXINON*^®^ lipoFIT assay (numares AG, Regensburg, Germany). Valine sample and reference sets were measured at a single site.

#### 2.8.3. Myo-Inositol 

In the absence of a valid reference method for myo-inositol measurement, trueness was evaluated by spike recovery experiments, as recommended in CLSI guidelines EP09-A3 and EP15-A3 [35,36]. A total of 150 human serum samples (4 mL each) were prepared from 2 to 3 single donor sera. For the lower concentration range, 44 of the pooled sera were dialyzed against 1× PBS supplemented with ≥10 mg/dL lactate, using Slida-A-Lyzer Mini Dialysis Devices with a molecular weight cutoff of 10,000 kDa (ThermoFisher Scientific, Waltham, MA, USA), which were then used for the desired low concentrations of myo-inositol. Each of the pooled human serum samples was divided into two aliquots. The first aliquot was used for mock-spiking with 5% volume of PBS to determine the mock levels of myo-inositol for the respective sample. The second set of aliquots was spiked with 5% volume of myo-inositol dissolved in PBS covering a concentration range from 0.02 to 0.40 mmol/L. Myo-inositol sample and reference sets were measured at a single site.

### 2.9. Sample Stability 

Sample stability experiments were conducted to evaluate its impact on the GFR_NMR_ equation according to the CLSI guideline EP25-A [37]. 

#### 2.9.1. Human Serum Specimen Storage Study (2–10 °C)

At baseline, blood was drawn from six donors using standard serum collection tubes with clotting activator without gel separator (Monovette-S neutral, Sarstedt, Nümbrecht, Germany). Samples were centrifuged within 2 hours of collection at 1800× *g* for 10 minutes to separate serum from the clot. Immediately after centrifugation, separated serum was used for the baseline measurements. The remaining human serum was dispensed into aliquots of 2 mL for each donor. These aliquots were stored at 2–10°C until they were prepared for analysis. For baseline measurements, five standard NMR samples were prepared immediately after centrifugation (t_0_) and measured within the first six hours after collection. For all of the other time points (day 1 to 8), 1 aliquot was taken per donor and used for the preparation and measurement of three NMR replicate samples. 

#### 2.9.2. NMR Sample Storage Study (on Board) 

For the storage study of NMR samples, the five NMR sample tubes prepared for the baseline measurement of the human serum storage study were stored in the sample changer of the device at 6 °C (±2 °C). The same NMR samples were repetitively measured at the selected time points (day 1, 4, 7, 9 and 10). 

### 2.10. Interfering Substances 

Interference testing was performed to evaluate its potential impact on the GFR_NMR_ equation according to CLSI guideline EP07 [38]. A total of 40 substances were identified to be clinically relevant and were tested for interference effects under ‘worst case’ conditions (Table S1). Per substance, 2 different serum pools with higher and lower GFR_NMR_ scores were prepared in 20 aliquots. Ten aliquots were spiked with the respective substance to the final concentration level as indicated in Table S1 and analyzed as test samples. The remaining ten aliquots were analyzed without spiked substance as control samples. Following this interference screen, a dose-response experiment was conducted for substances identified as potentially interfering with the GFR_NMR_, using different A (“high”) and B (“low”) serum pools. A total of 5 equidistant concentration levels were prepared by linear intermixture of a 100%-spiked test pool and a non-spiked control pool to generate and measure five replicate fractions containing 0%, 25%, 50%, 75% and 100% of the potentially interfering substance. 

### 2.11. Statistical Methods 

All of the calculations of performance evaluation and statistical tests were performed within R 4.0.2 [39]. Data structures were handled with the data.table Package [40]. Visualization was performed with packages lattice [41] and ggplot2 [42]. In general, calculations followed the recommendations of the respective CLSI guidelines briefly described as follows.

#### 2.11.1. Detection Capability

LoB, LoD and LoQ experimental data for creatinine, valine and myo-inositol were evaluated according to CLSI EP17-A2 [32]. LoB and LoD were determined depending on the distribution of the data for each parameter either by parametric or non-parametric method. For LoD calculation, the Cochran’s C-test [43] was applied to check the assumption that the variability of measurement results is consistent across low level samples [32]. In case Cochran’s *C* test failed, the “LoD Variant Approach: Nonparametric Analysis” was used (trial and error experiment design according to EP17-A2). LoQ for each reagent lot and parameter was calculated as minimum mean concentration of the corresponding pools that showed a within-laboratory precision with a coefficient of variation (CV%) < 20%. The final overall LoQ was defined as the maximum LoQ over all individual lots.

#### 2.11.2. Linearity

Linearity of creatinine, valine and myo-inositol NMR measurements was evaluated according to CLSI EP6-A [33]. The Pearson correlation coefficient [44] and repeatability (CV%) were calculated, and the maximum analytical concentration (upper limit of the linear range or LoL) was reported. Furthermore, polynomial regression analysis was performed for each analyte. For this, first-, second- and third-order polynomial models were built in the fashion of $y\;=b_{0}+b_{1}x$, $y\;=b_{0}+b_{1}x+b_{2}x^{2}$ and $y\;=b_{0}+b_{1}x+b_{2}x^{2}+b_{3}x^{3}$. A *t*-test was used to test whether non-linear coefficients (b_2_ and b_3_) were statistically significantly different (from 0). If no statistically significant terms were found (*p* > 0.05), then the data were considered as linear. If, however, a term was statistically significant (*p* < 0.05), then the degree of non-linearity was calculated for the best fitting polynomial according to standard error of regression. The linearity assessment for each analyte passed when a concentration range of at least five consecutive dilution levels had no more than 15% missing data, Pearson correlation coefficient *r* was ≥0.95, response appeared linear by visual inspection, repeatability (CV%) was <15%, and either no hint for non-linearity (no significant non-linear terms in 2nd or 3rd order polynomials) or a maximum relative degree of non-linearity <10% in case of detected non-linearity.

#### 2.11.3. Single-Site Precision 

Single-site precision was calculated for the creatinine, valine and myo-inositol analyte measurements, and for the GFR_NMR_ equation according to CLSI EP05-A3 [34]. All variance components (repeatability, between-run precision, between-day precision, and within-laboratory precision) were expressed as CV%. The acceptance criteria were that missing data per pool should not exceed 10% and that repeatability CV% should be ≤10% for GFR_NMR_, ≤12% for creatinine, and ≤20% for valine and myo-inositol.

#### 2.11.4. Multi-Site Precision 

Multi-site precision was calculated for the GFR_NMR_ equation according to CLSI EP05-A3 [34]. All variance components (repeatability, between-day precision, within-laboratory precision, and reproducibility) were expressed as CV%. Only pools according to the use case (for example, pool mean GFR_NMR_ scores <90 mL/min/1.73 m^2^) were analyzed. The acceptance criteria were that missing data per pool should be ≤10% and reproducibility CV% for GFR_NMR_ should be ≤10%.

#### 2.11.5. Method Comparison (Trueness)

Trueness of creatinine, valine and myo-inositol analyte measurements was expressed as the bias between the test measurement (three different sites) and a reference value, as described in CLSI EP09-A3 and EP15-A3 [35,36]. Data were analyzed using Passing-Bablok regression [45,46]. The estimated regression equation by Passing-Bablok regression, and the Pearson correlation coefficient between observed and expected values, were reported. The acceptance criteria were a Pearson correlation coefficient *r* ≥ 0.90 and a Passing-Bablok regression slope of 1.0 ± 0.15 (creatinine, myo-inositol) or 1.0 ± 0.075 (valine).

#### 2.11.6. Sample Stability

Sample stability was evaluated according to CLSI EP25-A [37]. Stability duration was evaluated for each condition and donor at the level of the GFR_NMR_ equation. Based on the regression analysis, the one-sided upper or lower 95% confidence interval of the regression line was determined. In case of a positive and statistically significant (*p* < 0.05) regression slope, the intersection of the 1-sided 95% confidence interval upper limit and the upper allowed drift limit defined the value of the stability duration. In case of a negative and statistically significant (*p* < 0.05) regression slope, the intersection of the 1-sided 95% confidence interval lower limit and the lower allowed drift limit defined the value of the stability duration. If the intersection was outside the defined period or if the regression slope was not statistically significant (*p* ≥ 0.05), the value of the stability duration was set to the maximum time point experimentally tested. Furthermore, the overall stability duration for each condition was defined as the minimum of stability durations over all donors given a maximum allowable measurement drift of 10%. Acceptance criteria were that missing data should be ≤10% per condition, and that initial time point (t_0_) as well as at least 2 additional time points having to be represented in the dataset.

#### 2.11.7. Interference

Interference testing was evaluated according to CLSI EP07 [38]. Interference evaluation followed a two-step process, an interference screen followed by a dose-response experiment, if applicable. For the interference testing evaluation, missing data should not exceed 10% per comparison (by pool, substance, and parameter). If the mean difference in the results of spiked and non-spiked samples (mean GFR_NMR_ in test minus mean GFR_NMR_ in control, across each pool) was >10% of the mean GFR_NMR_ in control (defined as mean relative bias), an interference effect by the tested substance was anticipated. In such case, a dose-response experiment was conducted using new serum pools containing 0%, 25%, 50%, 75% and 100% of spiked substance. Relative bias (GFR_NMR_ in test samples vs. mean GFR_NMR_ in control) was calculated and represented as strip plot, to determine the concentration at which interference occurred (relative bias > 10% of control). 

## 3. Results

**Figure 1:**
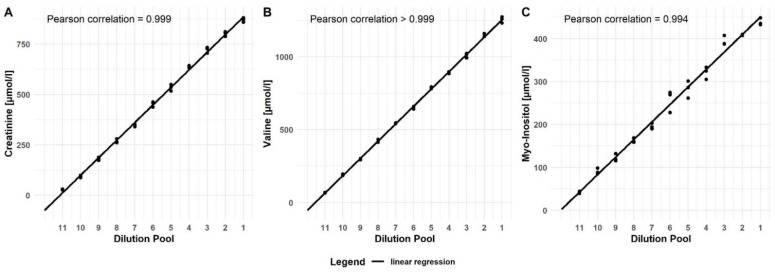
Linearity of NMR measurements for (**A**) creatinine, (**B**) valine, and (**C**) myo-inositol.

The analytical performance of the metabolite measurements by NMR and of the GFR_NMR_ equation was evaluated according to the respective Clinical and Laboratory Standards Institute (CLSI) guidelines. Detection capability (LoB, LoD, LoQ) of creatinine, valine, and myo-inositol measurements are shown in Table 1. Linearity of the NMR measurements was demonstrated, with a Pearson correlation coefficient *r* > 0.99 for the three metabolites (Figure 1). The linear analytical range (LoQ–LoL) was 25–870 µmol/L for creatinine, 30–1255 µmol/L for valine and 39–439 µmol/L for myo-inositol (Table 1).

The trueness of the creatinine, valine, and myo-inositol NMR measurements was evaluated by comparison to a reference method (creatinine, valine) or by spike recovery (myo-inositol). The Passing-Bablok regression equations and the Pearson correlation coefficients (*r*) between observed and expected values are shown in Table 1. The Passing-Bablok regression slope was ≤1.05 and *r* was ≥0.99 for the three metabolites.

Single-site, within-laboratory precision for creatinine, valine, and myo-inositol NMR measurements was calculated from nine different serum pools covering a broad concentration range and a total of 1080 measurements. Within-laboratory coefficients of variation (CV%) did not exceed 12.5%, 2.2% and 16.5% for creatinine, valine, and myo-inositol measurements, respectively (Table 1). Within-laboratory CV% were lower in the higher concentration ranges > 3-fold LoQ (≤6.6%, ≤2.2% and ≤6.4% for creatinine, valine, and myo-inositol, respectively) (Table 1).

**Figure 2:**
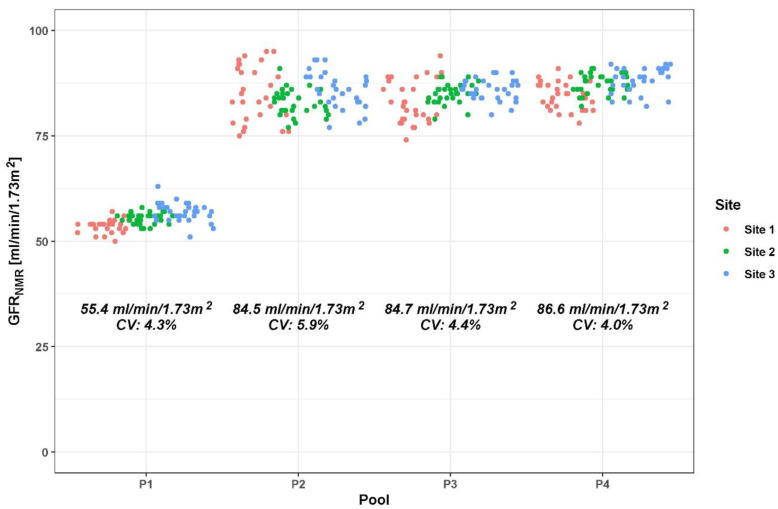
Scatterplot of multi-site precision (reproducibility) for the GFR_NMR_ equation calculated from 4 serum pools (P1–P4) with mean GFR_NMR_ scores below and above 60 mL/min/1.73 m^2^. Abbreviation: CV%, coefficient of variation.

Single-site, within-laboratory CV% of the GFR_NMR_ equation, calculated from 4 different serum pools with GFR_NMR_ results ranging from 53 to 82 mL/min/1.73 m^2^ and a total of 480 measurements, did not exceed 4.3%, with a maximum CV% for repeatability of 3.7% (Table 2). The inter-site reproducibility (between three sites), calculated from 4 serum pools ranging from 55 to 87 mL/min/1.73 m^2^ and 360 measurements, resulted in a maximum CV% of 5.9% (Table 2 and Figure 2).

**Figure 3:**
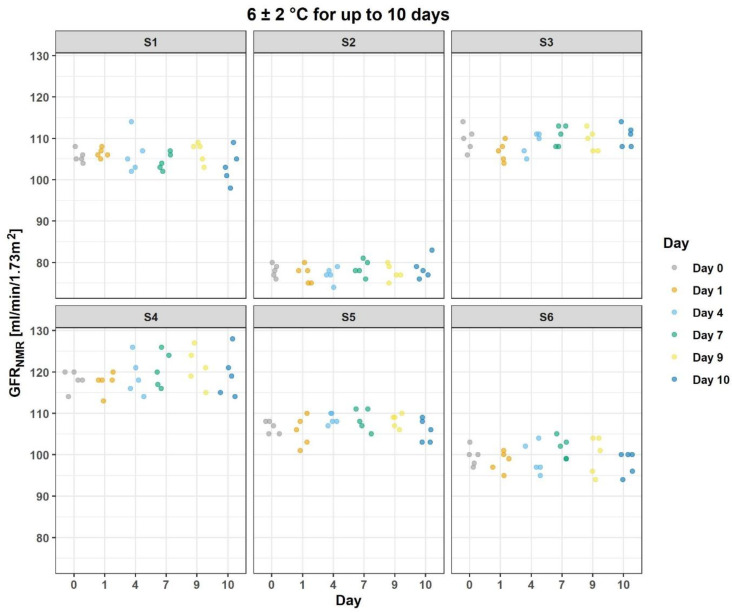
Scatterplot of GFR_NMR_ stability upon on-board storage at 6 ± 2°C of NMR samples for up to 10 days. The stability study was conducted using NMR samples prepared from the serum of six individual donors (S1–S6) and five replicate GFR_NMR_ measurements per time point.

The influence of storage of serum samples and of prepared NMR samples on the stability of the GFR_NMR_ results was evaluated using individual donor samples and sample storage conditions reflecting those commonly used in clinical practice. GFR_NMR_ stability was demonstrated for serum stored up to eight days at 2–10 °C and for NMR samples stored up to ten days on board of the NMR device at 6 ± 2 °C (Table 3 and Figure 3). Over the storage time duration investigated, the linear regression slope *p*-values were not significant (*p* > 0.05), except for on-board NMR sample with a slope of 0.29 mL/min/1.73 m^2^ per day, and a *p*-value of 0.043 (Table 3).

**Figure 4:**
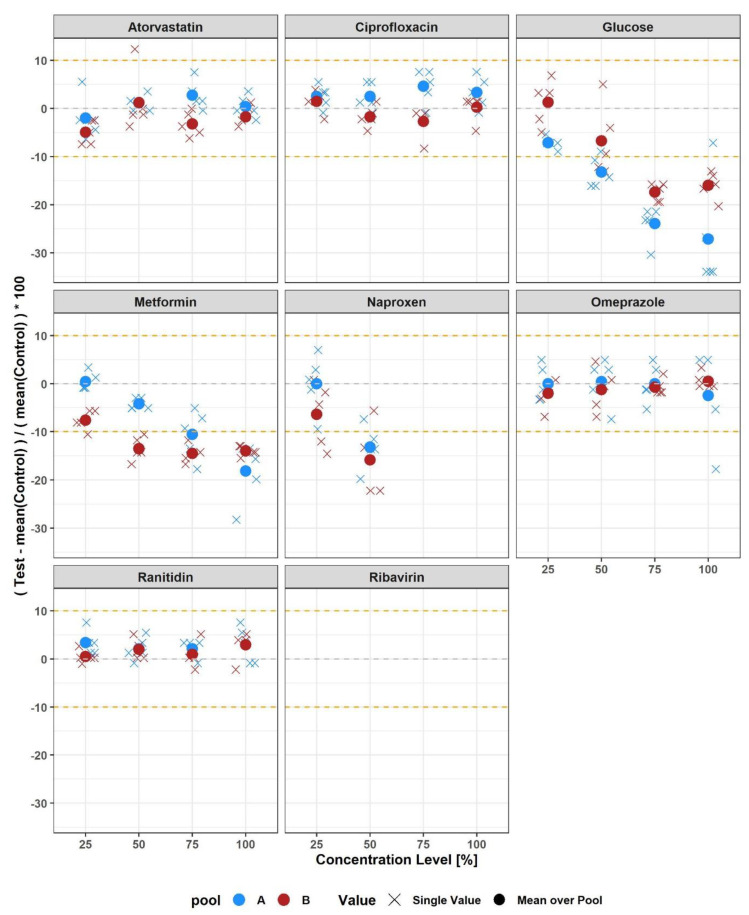
Dose−response interference experiments. Strip plots showing relative biases of GFR_NMR_ in serum pools (A: “low”, B: “high”, as defined in Table 4) spiked with increasing concentrations (0%, 25%, 50%, 75% and 100%) of the indicated potentially interfering substances (100% corresponding to the test concentration displayed in Table S1). Glucose > 13.9 mmol/L and metformin > 23.2 μmol/L caused falsely low GFR_NMR_ results, naproxen > 0.39 mmol/L caused falsely low or missing results at >0.78 mmol/L, and ribavirin ≥ 210 mg/L caused missing results. Interference suspected in the screening step (Table 4) was not confirmed in the dose-response assay for atorvastatin, ciprofloxacin, omeprazole and ranitidin.

Finally, we investigated a possible interference of the GFR_NMR_ assay by substances commonly expected to be present in clinical samples. Evaluated substances included endogenous metabolites indicative of clinically relevant metabolic disorders, such as ketone bodies, common dietary constituents such as caffeine, antihistamines, over-the-counter drugs such as cetirizine or ibuprofen, antibiotics such as amoxicillin or gentamicin, major diuretics such as furosemide or chlorothiazide, cholesterol-lowering drugs such as atorvastatin, or antidiabetic drugs such as glipizide or pioglitazone (Table S1). We screened for relevant potentially interfering substances under ‘worst-case’ conditions, for example at the highest concentration expected to be observed in clinical settings. In case of prescribed drugs, these concentrations were determined at approximately three times their therapeutic daily dose. In case of endogenous metabolites, physiologically very high concentrations with a suitable safety margin were chosen (Table S1). A total of 40 potentially interfering substances were each spiked into serum pools with higher and lower GFR_NMR_ scores (Table S2). Of 80 spiked sera, 9 presented a mean relative bias to unspiked sera >10% and 2 presented no GFR_NMR_ results (Table S2 and Table 4). These 11 affected sera involved eight substances, namely glucose, ciprofloxacin, atorvastatin, metformin, naproxen, omeprazole, ranitidin and ribavirin (Table 4), which were further evaluated in dose-response experiments using independent serum pools (Figure 4).

Dose-response experiments using concentrations of candidate interfering substances ranging from 25% to 100% of the concentration initially tested (Table S1) confirmed an interference for 4 substances: >13.9 mmol/L glucose, >23.2 μmol/L metformin, and >0.39 mmol/L naproxen caused falsely lower GFRNMR results, while >0.78 mmol/L naproxen and >210 mg/L ribavirin caused missing GFRNMR results (Figure 4). On the other hand, interference was not confirmed for atorvastatin, ciprofloxacin, omeprazole, and ranitidine (Figure 4). Thus, 4/40 (10.0%) tested substances showed some potentially interfering effect on the GFRNMR assay above certain serum levels.

## 4. Discussion

Laboratory testing for the evaluation of renal dysfunction includes estimation of glomerular filtration rate as the initial step. In this study, we describe the analytical performance validation of the standardized GFR_NMR_ assay, complementing its previously reported clinical validation [15]. We demonstrate GFR_NMR_ assay analytical performance as being compatible with its application in clinical routine settings, with linearity across a broad range of analyte concentrations, high precision, and comparability to reference methods, as well as stability ≥ 8 days under normal laboratory conditions. In direct comparison, detection capabilities of the NMR-based quantification of serum creatinine (LoQ of 25 µmol/L) were well below the 2.5%-tile of normal values of 45 µmol/L [47]. Imprecision of the creatinine quantification by NMR (CV% 3.4–12.5%) tended to be higher than that reported for conventional creatinine assays (CV% 0.4–4.4% for CREP2 Roche kit on Cobas c 503 based on IfU “2019-11, V 2.0 English”), but well below the 20% threshold recommended by the U.S. Food and Drug Administration. However, NMR technology allows the simultaneous detection of metabolite targets within a single analytical spectrum, resulting in a single level of impression for creatinine, valine, and myo-inositol as a biomarker constellation. As a direct consequence, the precision of GFR_NMR_, including repeatability and reproducibility, was similar to that of other eGFR methods [20,22,24,25], despite the higher level of imprecision for the individual biomarkers. Moreover, the reported imprecision in gold-standard measures of GFR contributes to an appreciable proportion of cases in which mGFR can differ by ≥30% [48]. The analytical validation study reported here thus demonstrates that when samples are adequately collected and processed, excellent analytical precision and accuracy is anticipated in clinical practice.

No analytical interference was observed for the majority (36/40 [90%]) of the relevant substances tested. Interferences were detected in only 4/40 (10%) of the investigated agents, resulting in underestimated GFR_NMR_ (for >13.9 mmol/L glucose, >23.2 μmol/L metformin, and >0.39 mmol/L naproxen) or loss of results (for >0.78 mmol/L naproxen and >210 mg/L ribavirin). Our results suggest that the presence of substances, including additive solutions and therapeutic agents, will have little impact in clinical practice on the results of the GFR_NMR_ equation. However, the situation is different for patients with diabetes mellitus with serum glucose levels above 13.9 mmol/L (250 mg/L). Fasting plasma glucose levels > 125 mg/dL (6.9 mmol/L) are considered indicative for diabetes [49]. Data from the National Health and Nutrition Examination Surveys 2005–2010 (NHANS) indicated fasting plasma glucose ranges of 89–177 mg/dL (4.94–9.82 mmol/L) in diabetic patients 20–44 years of age and 91–138 mg/dL (5.05–7.66 mmol/L) in patients ≥ 65 years of age [50]. Hence, in all cases, these values are well compatible with the observed interfering level of 13.9 mmol/L (250 mg/dL). Therefore, GFR_NMR_ testing after overnight fasting should be considered in diabetes management.

In addition, metformin is a glucose-lowering agent that is used as a first-line therapy for type 2 diabetes. Metformin is available in dosages of 500 mg, 850 mg and 1000 mg for oral administration to allow individualized blood glucose control. At usual clinical doses, metformin steady-state plasma concentrations are generally <1.5 μg/mL (11.6 µmol/L; [51]), for example less than 50% of the interference level obtained in our dose-response analysis. This makes a false-low GFR_NMR_ test result in vivo unlikely. Nevertheless, these results require further confirmation to verify, whether GFR_NMR_ underestimates GFR in patients with uncontrolled hyperglycemia on metformin treatment, as has been reported for Jaffé creatinine assays [52]. 

Naproxen >0.39 mmol/L interfered with GFR_NMR_. Naproxen is a common analgesic, antipyretic and anti-inflammatory drug, which inhibits the formation of prostaglandins by inhibiting hormone-sensitive lipase [53]. In our experiments, other common analgetic drugs such as ibuprofen, acetylsalicylic acid, and acetaminophen did not interfere with GFR_NMR_ testing. In clinical practice, they could therefore be substitutes to naproxen when GFR_NMR_ is planned. Alternatively, GFR_NMR_ testing could be delayed after withdrawal in patients acutely treated with naproxen to avoid false-low or missing results. 

In patients with or without CKD receiving active ribavirin treatment for hepatitis C, GFR_NMR_ should be replaced by alternative test methods to minimize the risk of missing test results with GFR_NMR_.

Our study presents minimal limitations. First, we used samples from healthy donors only for the analytical validation analysis, thus not reflecting the lower range of eGFR that would be expected from patients with progressed CKD. However, we generated samples simulating conditions of patients with low eGFR, as recommended by CLSI. The expansion of the interference experiments by evaluating in-vivo samples of patients with a high likelihood for the presence of certain interfering substances, as shown by others [23], would have complemented our approach. This limitation was however compensated by the analysis of ‘worst-case’ scenarios and the analysis of a high number of substances, optimizing the probability of identifying putative interfering substances. Finally, in this analytical performance study, the impact of the cystatin C measuring method (Tina-Quant Cystatin C Gen.2 assay, Roche) was not considered for the analytical performance evaluation of GFR_NMR_, which might introduce a bias in the evaluation. However, this is unlikely‘ given that the cystatin C assay was conducted with the recommended ERM-DA471/IFCC calibrator [29,54], and that its excellent analytical performance is well documented [27,28,54,55].

A strength of our analytical validation study is that it closely followed the recommendations of the CLSI guidelines, and that samples were stored and handled uniformly across the study. Moreover, our interference study included a large number (n = 40) of relevant potentially interfering agents, which contrasts with most reported analytical validation studies [20,23,24,56].

## 5. Conclusions

Developing a robust assay with fully characterized analytical properties is a critical step toward the implementation of a reliable and accurate biomarker-based diagnostic GFR assay into routine clinical practice. Here, we demonstrate an excellent analytical performance of the GFR_NMR_ assay, complementing its recently reported superior clinical performance compared to existing eGFR equations [15]. In regards to routine clinical practice, we implemented the GFR_NMR_ assay on an NMR platform without the need for pre-separation of analytes before quantification. Such an optimized process allows expedited sample processing with limited opportunities for human operator error, all of which make the GFR_NMR_ assay an attractive option for a reliable diagnostic test that can be run in a decentralized manner.

## 6. Patents

ES has a patent application WO002020065092A1 pending.

## Figures and Tables

**Table 1:** Detection capability, upper limit of linear range, trueness and single-site precision evaluation of NMR measurements for creatinine, valine and myo-inositol.

	Creatinine		Valine		Myo-Inositol	
**LoB [µmol/L]**	20		20		39	
**LoD [µmol/L]**	25		26		39	
**LoQ [µmol/L]**	25		30		39	
**LoL [µmol/L]**	<870		<1255		<439	
**Trueness PB; *r***	y = 1.028x−5.364; 0.990		y = 1.050x + 0.450; 0.996		y = 1.002x + 2.269; 0.990	
**Precision ^1^**						
**Pool**	**Mean; SD [µmol/L]**	**CV%**	**Mean; SD [µmol/L]**	**CV%**	**Mean; SD [µmol/L]**	**CV%**
1	42.8; 5.4	12.5	211.4; 5.2	2.2	57.3; 4.4	14.6
2	62.8; 3.4	5.4	231.9; 5.4	2.1	63.4; 4.8	16.5
3	69.9; 5.4	7.8	280.2; 5.4	2.1	67.7; 5.2	14.3
4	76.0; 3.2	4.2	298.7; 4.6	2.0	69.1; 3.2	15.0
5	78.4; 5.2	6.6	315.3; 3.2	1.9	72.0; 3.4	15.8
6	85.4; 4.4	5.2	354.0; 3.4	2.1	80.4; 5.4	13.9
7	107.5; 4.8	4.5	383.5; 4.4	2.0	88.6; 4.6	11.7
8	123.8; 4.6	3.7	406.3; 5.9	2.2	204.6; 5.9	6.4
9	173.7; 5.9	3.4	463.8; 4.8	2.2	224.0; 5.4	5.9

^1^ Single-site within-laboratory precision (1 site × 9 serum pools × 3 replicates per pool × 2 runs per day × 20 days; N = 1080 measurements). The nine serum pools were chosen to cover low to high physiological concentrations of the respective metabolites. Abbreviations: LoB, limit of blank; LoD, limit of detection; LoQ, limit of quantification; LoL, upper limit of linear range; *r*, Pearson correlation coefficient; PB, Passing-Bablok regression; CV%, coefficient of variation (for within-laboratory precision) (%).

**Table 2:** GFR_NMR_ single- and multi-site precision.

Precision	N	Pool	Mean [mL/min/1.73 m^2^]	Repeatability [CV%]	Between-Run [CV%]	Between-Day [CV%]	Within-Laboratory [CV%]	Reproduci-bility [CV%]
Single-site ^1^	480	P1	53.5	2.8	1.3	2.4	3.9	n.a.
		P2	55.4	2.9	1.8	0.0	3.4	n.a.
		P3	78.1	3.7	0.0	1.2	3.9	n.a.
		P4	82.4	3.6	2.3	1.0	4.3	n.a.
Multi-site ^2^	360	P1	55.4	3.3	n.a.	0.3	3.3	4.3
		P2	84.5	5.1	n.a.	2.8	5.8	5.9
		P3	84.7	2.9	n.a.	3.3	4.4	4.4
		P4	86.6	2.8	n.a.	2.1	3.5	4.0

^1^ 1 site × 4 serum pools × 3 replicates per pool × 2 runs per day × 20 days (N = 480 measurements); ^2^ 3 sites × 4 serum pools × 6 replicates per pool × 1 run per day × 5 days (N = 360 measurements). Abbreviations: CV%, coefficient of variation; n.a., not applicable.

**Table 3:** GFR_NMR_ score stability during storage of human serum (2–10°C) and of prepared NMR samples (6 ± 2°C), relative to mean GFR_NMR_ at day 0 (t_0_).

Stability	Donor	Mean GFR_NMR_ at t_0_ [mL/min/1.73 m^2^]	Slope [mL/min/1.73 m^2^]	Intercept [mL/min/1.73 m^2^]	Slope *p*-Value	Duration [days] ^2^
Serum samples (2–10°C) ^1^	1	105.6	−0.14	102.65	0.874	8
	2	78.0	−0.38	76.88	0.492	8
	3	109.8	−1.08	108.36	0.164	8
	5	106.6	−0.35	103.57	0.647	8
	6	99.6	0.21	97.03	0.723	8
On-board NMR samples (6 ± 2 °C)	1	105.6	−0.16	106.23	0.310	10
	2	78.0	0.08	77.42	0.327	10
	3	109.8	0.21	108.28	0.196	10
	4	118.0	0.29	117.77	0.043	10
	5	106.6	0.09	106.76	0.618	10
	6	99.6	0.03	99.26	0.865	10

^1^ Volume of serum of Donor 4 was insufficient to cover all serum stability time points and was excluded from the analysis; ^2^ Calculated stability duration, as defined in Materials and Methods (2.11.6.).

**Table 4:** Potentially interfering substances with mean relative bias >± 10% in at least one serum pool in the interference screen.

Substance ^1^	Pool ^2^	Mean Relative Bias [%]
Atorvastatin	High	−12.20
Ciprofloxacin	High	+10.81
	Low	+11.84
Glucose	High	−23.34
	Low	−26.70
Metformin	High	−10.48
Naproxen	Low	−17.41
Omeprazole	High	−15.37
Ranitidin	High	−15.10
Ribavirin	High	No result
	Low	No result

^1^ See Table S1 for tested concentrations; ^2^ Two serum pools with higher (“High”) and lower (“Low”) GFR_NMR_ values were tested per interference assay.

## Data Availability

The data presented in this study are available within the article or Supplementary Materials.

## Supplementary Materials

The following supporting information can be downloaded at: https://www.mdpi.com/article/10.3390/diagnostics12051120/s1, Table S1: Substances selected for interference study with information about supplier, substance batch, final test concentration and comments on the substance clinical background; Table S2: Interference study results for substances described in Table S1, showing the mean GFR_NMR_ values for control and test pools and the relative bias for serum pools with higher and lower GFR_NMR_ scores.

## References

[1] Lv J.-C., Zhang L.-X. (2019). Prevalence and Disease Burden of Chronic Kidney Disease. Adv. Exp. Med. Biol..

[2] National Kidney Foundation (2002). K/DOQI clinical practice guidelines for chronic kidney disease: Evaluation, classification, and stratification. Am. J. Kidney Dis. Off. J. Natl. Kidney Found..

[3] Glassock R.J., Warnock D.G., Delanaye P. (2017). The global burden of chronic kidney disease: Estimates, variability and pitfalls. Nat. Rev. Nephrol..

[4] CKD Evaluation and Management—KDIGO. https://kdigo.org/guidelines/ckd-evaluation-and-management/.

[5] (2013). KDIGO 2012 Clinical Practice Guideline for the Evaluation and Management of Chronic Kidney Disease. Kidney Int. Suppl..

[6] Smith W.H. (1951). Measurement of the filtration rate. The Kidney—Structure and Function in Health and Disease.

[7] Soveri I., Berg U.B., Björk J., Elinder C.-G., Grubb A., Mejare I., Sterner G., Bäck S.-E. (2014). SBU GFR Review Group Measuring GFR: A systematic review. Am. J. Kidney Dis. Off. J. Natl. Kidney Found..

[8] Seegmiller J.C., Eckfeldt J.H., Lieske J.C. (2018). Challenges in Measuring Glomerular Filtration Rate: A Clinical Laboratory Perspective. Adv. Chronic Kidney Dis..

[9] Hsu C., Bansal N. (2011). Measured GFR as “Gold Standard”—All that Glitters Is Not Gold?. Clin. J. Am. Soc. Nephrol..

[10] Topf J.M., Inker L.A., Lerma E.V., Sparks M.A., Topf J. (2019). Chapter 3—Measurement of glomerular filtration rate. Nephrology Secrets.

[11] Levey A.S., Stevens L.A., Schmid C.H., Zhang Y.L., Castro A.F., Feldman H.I., Kusek J.W., Eggers P., Van Lente F., Greene T. (2009). A new equation to estimate glomerular filtration rate. Ann. Intern. Med..

[12] Levey A.S., Tighiouart H., Inker L.A. (2021). Improving Glomerular Filtration Rate Estimation-Across the Age and Diversity Spectrum. Ann. Intern. Med..

[13] Inker L.A., Schmid C.H., Tighiouart H., Eckfeldt J.H., Feldman H.I., Greene T., Kusek J.W., Manzi J., Van Lente F., Zhang Y.L. (2012). Estimating glomerular filtration rate from serum creatinine and cystatin C. N. Engl. J. Med..

[14] Pottel H., Björk J., Courbebaisse M., Couzi L., Ebert N., Eriksen B.O., Dalton R.N., Dubourg L., Gaillard F., Garrouste C. (2021). Development and Validation of a Modified Full Age Spectrum Creatinine-Based Equation to Estimate Glomerular Filtration Rate: A Cross-sectional Analysis of Pooled Data. Ann. Intern. Med..

[15] Stämmler F., Grassi M., Meeusen J.W., Lieske J.C., Dasari S., Dubourg L., Lemoine S., Ehrich J., Schiffer E. (2021). Estimating Glomerular Filtration Rate from Serum Myo-Inositol, Valine, Creatinine and Cystatin C. Diagnostics.

[16] Steubl D., Inker L.A. (2018). How best to estimate glomerular filtration rate? Novel filtration markers and their application. Curr. Opin. Nephrol. Hypertens..

[17] Porrini E., Ruggenenti P., Luis-Lima S., Carrara F., Jiménez A., de Vries A.P.J., Torres A., Gaspari F., Remuzzi G. (2019). Estimated GFR: Time for a critical appraisal. Nat. Rev. Nephrol..

[18] Delanaye P., Cavalier E., Cristol J.-P., Delanghe J.R. (2014). Calibration and precision of serum creatinine and plasma cystatin C measurement: Impact on the estimation of glomerular filtration rate. J. Nephrol..

[19] Kuster N., Cristol J.-P., Cavalier E., Bargnoux A.-S., Halimi J.-M., Froissart M., Piéroni L., Delanaye P. (2014). Société Française de Biologie Clinique (SFBC) Enzymatic creatinine assays allow estimation of glomerular filtration rate in stages 1 and 2 chronic kidney disease using CKD-EPI equation. Clin. Chim. Acta Int. J. Clin. Chem..

[20] Delanaye P., Pieroni L., Abshoff C., Lutteri L., Chapelle J.-P., Krzesinski J.-M., Hainque B., Cavalier E. (2008). Analytical study of three cystatin C assays and their impact on cystatin C-based GFR-prediction equations. Clin. Chim. Acta Int. J. Clin. Chem..

[21] Coresh J., Astor B.C., McQuillan G., Kusek J., Greene T., Van Lente F., Levey A.S. (2002). Calibration and random variation of the serum creatinine assay as critical elements of using equations to estimate glomerular filtration rate. Am. J. Kidney Dis. Off. J. Natl. Kidney Found..

[22] Piéroni L., Delanaye P., Boutten A., Bargnoux A.-S., Rozet E., Delatour V., Carlier M.-C., Hanser A.-M., Cavalier E., Froissart M. (2011). A multicentric evaluation of IDMS-traceable creatinine enzymatic assays. Clin. Chim. Acta Int. J. Clin. Chem..

[23] Greenberg N., Roberts W.L., Bachmann L.M., Wright E.C., Dalton R.N., Zakowski J.J., Miller W.G. (2012). Specificity characteristics of 7 commercial creatinine measurement procedures by enzymatic and Jaffe method principles. Clin. Chem..

[24] Freed T.A., Coresh J., Inker L.A., Toal D.R., Perichon R., Chen J., Goodman K.D., Zhang Q., Conner J.K., Hauser D.M. (2019). Validation of a Metabolite Panel for a More Accurate Estimation of Glomerular Filtration Rate Using Quantitative LC-MS/MS. Clin. Chem..

[25] Tan G.D., Lewis A.V., James T.J., Altmann P., Taylor R.P., Levy J.C. (2002). Clinical usefulness of cystatin C for the estimation of glomerular filtration rate in type 1 diabetes: Reproducibility and accuracy compared with standard measures and iohexol clearance. Diabetes Care.

[26] Murthy K., Stevens L.A., Stark P.C., Levey A.S. (2005). Variation in the serum creatinine assay calibration: A practical application to glomerular filtration rate estimation. Kidney Int..

[27] Kyhse-Andersen J., Schmidt C., Nordin G., Andersson B., Nilsson-Ehle P., Lindström V., Grubb A. (1994). Serum cystatin C, determined by a rapid, automated particle-enhanced turbidimetric method, is a better marker than serum creatinine for glomerular filtration rate. Clin. Chem..

[28] Newman D.J., Thakkar H., Edwards R.G., Wilkie M., White T., Grubb A.O., Price C.P. (1995). Serum cystatin C measured by automated immunoassay: A more sensitive marker of changes in GFR than serum creatinine. Kidney Int..

[29] Grubb A., Blirup-Jensen S., Lindström V., Schmidt C., Althaus H., Zegers I. (2010). IFCC Working Group on Standardisation of Cystatin C (WG-SCC). First certified reference material for cystatin C in human serum ERM-DA471/IFCC. Clin. Chem. Lab. Med..

[30] Ehrich J., Dubourg L., Hansson S., Pape L., Steinle T., Fruth J., Höckner S., Schiffer E. (2021). Serum Myo-Inositol, Dimethyl Sulfone, and Valine in Combination with Creatinine Allow Accurate Assessment of Renal Insufficiency-A Proof of Concept. Diagnostics.

[31] Teipel J.C., Hausler T., Sommerfeld K., Scharinger A., Walch S.G., Lachenmeier D.W., Kuballa T. (2020). Application of 1H Nuclear Magnetic Resonance Spectroscopy as Spirit Drinks Screener for Quality and Authenticity Control. Foods.

[32] Clinical & Laboratory Standards Institute EP17-A2: Evaluation of Detection Capability for Clinical Laboratory Measurement Procedures; Approved Guideline—Second Edition. https://clsi.org/standards/products/method-evaluation/documents/ep17/.

[33] Clinical & Laboratory Standards Institute EP6-A: Evaluation of the Linearity of Quantitative Measurement Procedures—A Statistical Approach; Approved Guideline. https://clsi.org/standards/products/method-evaluation/documents/ep06/.

[34] Clinical & Laboratory Standards Institute EP05-A3: Evaluation of Precision of Quantitative Measurement Procedures; Approved Guideline—Third Edition. https://clsi.org/standards/products/method-evaluation/documents/ep05/.

[35] Clinical & Laboratory Standards Institute EP09-A3: Measurement Procedure Comparison and Bias Estimation Using patient Samples; Approved Guideline—Third Edition. https://clsi.org/standards/products/method-evaluation/documents/ep09/.

[36] Clinical & Laboratory Standards Institute EP15-A3: User Verification of Precision and Estimation of Bias; Approved Guideline—Third Edition. https://clsi.org/standards/products/method-evaluation/documents/ep15/.

[37] Clinical & Laboratory Standards Institute EP25-A: Evaluation of Stability of In Vitro Diagnostic Reagents; Approved Guideline. https://clsi.org/standards/products/method-evaluation/documents/ep25/.

[38] Clinical & Laboratory Standards Institute EP07: Interference Testing in Clinical Chemistry—Third Edition. https://clsi.org/standards/products/method-evaluation/documents/ep07/.

[39] (2020). R Core Team. R: The R Project for Statistical Computing, R Package Version 4.0.2. https://www.r-project.org/.

[40] Dowle M., Srinivasan A., Gorecki J., Chirico M., Stetsenko P., Short T., Lianoglou S., Antonyan E., Bonsch M., Parsonage H. (2020). Data.table: Extension of “Data.frame”; R Package Version 1.13.2. https://CRAN.R-project.org/package=data.table.

[41] Sarkar D. (2008). Lattice: Multivariate Data Visualization with R.

[42] Wickham H. (2016). ggplot2: Elegant Graphics for Data Analysis.

[43] Cochran W.G. (1941). The Distribution of the Largest of a Set of Estimated Variances as a Fraction of Their Total. Ann. Eugen..

[44] Freedman D., Pisani R., Purves R. (2007). Pearson Correlation. Statistics, 4th Edition International Student Edition.

[45] Passing H., Bablok, null A new biometrical procedure for testing the equality of measurements from two different analytical methods (1983). Application of linear regression procedures for method comparison studies in clinical chemistry, Part I. J. Clin. Chem. Clin. Biochem. Z. Klin. Chem. Klin. Biochem..

[46] Passing H., Bablok W. (1984). Comparison of several regression procedures for method comparison studies and determination of sample sizes. Application of linear regression procedures for method comparison studies in Clinical Chemistry, Part II. J. Clin. Chem. Clin. Biochem. Z. Klin. Chem. Klin. Biochem..

[47] Shimada M., Dass B., Ejaz A.A., Assessment of Elevated Creatinine—Differential Diagnosis of Symptoms BMJ Best Practice. https://bestpractice.bmj.com/topics/en-gb/935.

[48] Kwong Y.-T.D., Stevens L.A., Selvin E., Zhang Y.L., Greene T., Van Lente F., Levey A.S., Coresh J. (2010). Imprecision of urinary iothalamate clearance as a gold-standard measure of GFR decreases the diagnostic accuracy of kidney function estimating equations. Am. J. Kidney Dis. Off. J. Natl. Kidney Found..

[49] American Diabetes Association Professional Practice Committee 2 (2021). Classification and Diagnosis of Diabetes: Standards of Medical Care in Diabetes—2022. Diabetes Care.

[50] Menke A., Knowler W.C., Cowie C.C., Cowie C.C., Casagrande S.S., Menke A., Cissell M.A., Eberhardt M.S., Meigs J.B., Gregg E.W., Knowler W.C., Barrett-Connor E., Becker D.J. (2018). Physical and Metabolic Characteristics of Persons with Diabetes and Prediabetes. Diabetes in America.

[51] Sutkowska E., Fortuna P., Wisniewski J., Sutkowska K., Hodurek P., Gamian A., Kaluza B. (2021). Low metformin dose and its therapeutic serum concentration in prediabetes. Sci. Rep..

[52] den Elzen W.P.J., Cobbaert C.M., Klein Gunnewiek J.M.T., Bakkeren D.L., van Berkel M., Frasa M.A.M., Herpers R.L.J.M., Kuypers A.W.H.M., Ramakers C., Roelofsen-de Beer R.J.A.C. (2018). Glucose and total protein: Unacceptable interference on Jaffe creatinine assays in patients. Clin. Chem. Lab. Med..

[53] Taha M.O., Dahabiyeh L.A., Bustanji Y., Zalloum H., Saleh S. (2008). Combining ligand-based pharmacophore modeling, quantitative structure-activity relationship analysis and in silico screening for the discovery of new potent hormone sensitive lipase inhibitors. J. Med. Chem..

[54] FDA (2014). 510(k) Summary for Tina-Quant Cystatin C Gen.2 Assay, Roche Diagnostics. https://fda.report/PMN/K080811/8/K080811.pdf.

[55] Hansson L.-O., Grubb A., Lidén A., Flodin M., Berggren A., Delanghe J., Stove V., Luthe H., Rhode K.-H., Beck C. (2010). Performance evaluation of a turbidimetric cystatin C assay on different high-throughput platforms. Scand. J. Clin. Lab. Investig..

[56] Matyus S.P., Braun P.J., Wolak-Dinsmore J., Saenger A.K., Jeyarajah E.J., Shalaurova I., Warner S.M., Fischer T.J., Connelly M.A. (2015). HDL particle number measured on the Vantera^®^, the first clinical NMR analyzer. Clin. Biochem..

