# Supplementary material for: Analytical Validation of GFRNMR: A Blood-Based Multiple Biomarker Assay for Accurate Estimation of Glomerular Filtration Rate
Source: Diagnostics (Basel). 2022 Apr 30;12(5):1120. doi: 10.3390/diagnostics12051120 (PMC9139323; doi:10.3390/diagnostics12051120)
Supplement: Supplementary file 1 [file diagnostics-12-01120-s001.zip › diagnostics-1692311-supplementary.pdf]

Supplementary Materials

Table S1. Substances selected for interference study with information about supplier, substance batch, final test concentration and comments on the substance clinical background.

Substance	Supplier	Batch no.	Test concentration	Comment (clinical background)
Acetaminophen	Sigma-Aldrich	SLCB2770	1.03 mmol/L	Analgesic, common over the counter drug
Acetoacetic acid	Sigma-Aldrich	SLBX9935	2.0 mmol/L	Endogen, Ketonemia/ketonuria; increased blood concentration
Acetone	TCI (Tokyo Chemical Industry)	Z4QXF-BX	17 mmol/L	Endogen, Ketonemia/ketonuria; increased blood concentration
Acetylcysteine	Sigma-Aldrich	WXBC3104V	920 µmol/L	Cold remedy, common over the counter drug
Acetylsalicylic acid	TCI (Tokyo Chemical Industry)	QATRA-OF	167 µmol/L	Analgesic, common over the counter drug
Amoxicillin	Sigma-Aldrich	106M4843V	148 µmol/L	Antibiotic, common prescription drug
Atorvastatin	Sigma-Aldrich	LRAA9204	1.34 µmol/L	Statin, common prescription drug
Azathioprine	Sigma-Aldrich	LRAA9079	9.31 µmol/L	Immunosuppressant, standard therapy
Bilirubin (conjug.)	EMD Millipore Corp	3429119	475 µmol/L	Endogen, integral part of serum
Caffeine	Sigma-Aldrich	BCBW9292	556 µmol/L	Stimulant, common in beverages (coffee, tea, etc.)
Ceftriaxone	Sigma-Aldrich	LRAA9170	1.51 mmol/L	Antibiotic, common prescription drug
Cetirizine	Sigma-Aldrich	BCCD6341	11.2 µmol/L	Antihistamine, common over the counter drug
Chlorothiazide	TCI (Tokyo Chemical Industry)	KA6XA-FM	91.3 µmol/L	Diuretic and antihypertensive, common prescription drug
Chlorpropamide	Alfa Aesar	61400077	2.38 mmol/L	Antidiabetic, common prescription drug
Cimetidine	Alfa Aesar	R30F011	119 µmol/L	H2 receptor antagonist, common prescription drug
Ciprofloxacin	TCI (Tokyo Chemical Industry)	SOBSA-MI	36.2 µmol/L	Antibiotic, common prescription drug
Cyclosporin A	Cayman Chemicals	0516149-15	1080 mg/L (1.5 µmol/L)	Immunosuppressant, standard therapy
Enalaprilat	TCI (Tokyo Chemical Industry)	BFVVH-FJ	2.35 µmol/L	ACE inhibitor, common prescription drug
Fenofibrate	TCI (Tokyo Chemical Industry)	VJ5HF-PI	125 µmol/L	Hypolipidemic agent, common prescription drug
Furosemide	Sigma-Aldrich	MKCM6678	48.1 µmol/L	Diuretic, common prescription drug
Gentamicin	Sigma-Aldrich	110726	62.8 µmol/L	Antibiotic, common prescription drug
Glipizide	Sigma-Aldrich	MKBW7617V	6.73 µmol/L	Antidiabetic, common prescription drug

Glucose	TCI (Tokyo Chemical Industry)	UN33L-LT	55.5 mmol/L	Endogen, integral part of serum
Hemoglobin	Sigma-Aldrich	SLBR9638V	10 g/L	Endogen, integral part of serum
β-Hydroxybutyric acid	Sigma-Aldrich	BCBF1544V	7.7 mmol/L	Endogen, Ketonemia/ketonuria; increased blood concentration
Ibuprofen	Cayman Chemicals	0437675-71	1.06 mmol/L	Analgesic, common over the counter drug
Metformin	Cayman Chemicals	0544112-30	92.9 µmol/L	Antidiabetic, common prescription drug
Metoprolol	Sigma-Aldrich	H0L334	5.61 µmol/L	Beta blocker, common prescribed drug in Germany
Naproxen	Alfa Aesar	X23F019	1.56 mmol/L	Analgesic, common over the counter drug
Nifedipine	Cayman Chemicals	0515911-12	1.7 µmol/L	Calcium channel blocker, common prescription drug
Omeprazole	Sigma-Aldrich	LRAB0280	24.3 µmol/L	Proton pump inhibitor, common prescription drug
Pioglitazone (hydrochloride)	Sigma-Aldrich	LRAC4119	13.4 µmol/L	Antidiabetic, common prescription drug
Propranolol	Sigma-Aldrich	BCBQ4523V	3.88 µmol/L	Beta blocker, common prescription drug
Ranitidin	Sigma-Aldrich	MKCC2059	33.4 µmol/L	Antihistamine, standard therapy
Ribavirin	Cayman Chemicals	0470394-10	840 mg/L	Antiviral, standard therapy
Rifampicin	Sigma-Aldrich	MKCC2435	58.3 µmol/L	Antibiotic, common prescription drug
Salicylic acid	Sigma-Aldrich	BCBT7132	207 µmol/L	Metabolite of acetylsalicylic acid
Spironolactone	Sigma-Aldrich	MKBZ9531V	1.33 µmol/L	Aldosterone antagonist, common prescription drug
Trimethoprim	Alfa Aesar	N10G008	145 µmol/L	Antibiotic, common prescription drug
Valsartan	Sigma-Aldrich	LRAA9111	26.9 µmol/L	AT1-Antagonist, common prescription drug

Table S2. Interference study results for substances described in Table S1, showing the mean GFR_{NMR} values for control and test pools and the relative bias for serum pools with higher and lower GFR_{NMR} scores.

Substance [1]	Pool [2]	Control		Test		Relative Bias [3] [%]
		Measurements (N)	Mean [ml/min/1.73m²]	Measurements (N)	Mean [ml/min/1.73m²]	
Acetaminophen	High	10	73.5	10	71.2	-3.13
	Low	10	53.8	10	54.3	0.93
Acetoacetic acid	High	10	71.4	10	73.5	2.94
	Low	10	57.1	10	57.7	1.05
Acetone	High	10	73.5	10	73.0	-0.68
	Low	10	54.6	10	53.9	-1.28
Acetylcysteine	High	10	110.3	10	102.8	-6.80
	Low	10	56.2	10	59.4	5.69
Acetylsalicylic Acid	High	10	100.7	10	104.7	3.97
	Low	10	61.8	10	62.4	0.97
Amoxicillin	High	10	110.3	10	113.9	3.26
	Low	10	56.2	10	55.3	-1.60
Atorvastatin	High	9	68.3	7	60.0	-12.20
	Low	10	60.6	10	59.1	-2.48
Azathioprin	High	9	68.3	6	63.8	-6.59
	Low	10	60.6	10	56.9	-6.11
beta Hydroxybutyrate	High	10	110.3	10	115.0	4.26
	Low	10	56.2	10	55.7	-0.89
Bilirubin	High	10	71.4	10	72.1	0.98
	Low	10	59.0	10	58.7	-0.51
Caffeine	High	10	71.4	10	71.3	-0.14
	Low	10	58.8	10	58.5	-0.51
Ceftriaxone	High	10	114.9	10	107.1	-6.79
	Low	10	55.5	10	53.5	-3.60
Cetirizine	High	10	114.9	10	121.5	5.74
	Low	10	55.5	10	59.1	6.49
Chlorothiazide	High	10	110.3	10	111.4	1.00
	Low	10	56.2	10	55.4	-1.42
Chlorpropamide	High	10	110.3	10	114.0	3.35
	Low	10	56.2	10	60.9	8.36
Cimetidine	High	10	121.6	10	127.5	4.85
	Low	10	47.9	10	47.0	-1.88
Ciprofloxacin	High	10	114.9	10	128.5	11.84
	Low	10	55.5	10	61.5	10.81
Cyclosporin	High	10	73.9	10	73.7	-0.27
	Low	10	62.6	10	62.6	0.00
Enalaprilat	High	10	73.9	10	73.7	-0.27
	Low	10	62.6	10	62.4	-0.32
Fenofibrate	High	10	73.9	10	73.8	-0.14
	Low	10	62.6	10	63.4	1.28
Furosemide	High	10	73.9	10	72.0	-2.57
	Low	10	62.6	10	63.5	1.44

Gentamicin	High	10	80.1	10	78.8	-1.62
	Low	10	51.4	10	49.1	-4.47
Glipizide	High	10	73.9	10	74.5	0.81
	Low	10	62.6	9	64.2	2.59
Glucose	High	10	100.7	10	77.2	-23.34
	Low	10	61.8	10	45.3	-26.70
Hemoglobin	High	10	73.9	10	79.8	7.98
	Low	10	62.6	10	65.6	4.79
Ibuprofen	High	10	87.4	10	93.7	7.21
	Low	10	48.3	10	47.9	-0.83
Metformin	High	10	73.5	10	65.8	-10.48
	Low	10	53.8	10	55.7	3.53
Metoprolol	High	10	73.5	10	73.7	0.27
	Low	10	53.8	10	53.8	0.00
Naproxen	High	10	82.2	10	89.7	9.12
	Low	10	73.5	10	60.7	-17.41
Nifedipine	High	10	77.9	10	77.3	-0.77
	Low	10	50.1	10	47.8	-4.59
Omeprazole	High	10	73.5	10	62.2	-15.37
	Low	10	53.8	10	53.4	-0.74
Pioglitazone	High	10	87.4	10	87.0	-0.46
	Low	10	48.3	10	48.5	0.41
Propanolol	High	10	100.7	10	101.3	0.60
	Low	10	61.8	10	62.5	1.13
Ranitidin	High	10	73.5	10	62.4	-15.10
	Low	10	53.8	10	53.6	-0.37
Ribavirin	High	10	121.6	0	No result	N/A
	Low	10	47.9	0	No result	N/A
Rifampicin	High	10	87.4	10	86.1	-1.49
	Low	10	48.3	10	47.7	-1.24
Salicylic acid	High	10	73.5	10	71.9	-2.18
	Low	10	53.8	10	54.7	1.67
Spironolactone	High	9	68.3	7	63.1	-7.60
	Low	10	60.6	10	55.2	-8.91
Trimethoprim	High	10	87.9	10	86.2	-1.93
	Low	10	47.6	10	48.7	2.31
Valsartan	High	9	68.3	6	68.2	-0.24
	Low	10	60.6	10	54.9	-9.41

[1] See Table S1 for tested concentrations; [2] Two serum pools with higher ("High") and lower ("Low") GFR_{NMR} values were tested per interference assay; [3] Relative bias calculated as the mean relative difference in the results of spiked (Test) and non-spiked (Control) samples (mean GFR_{NMR} in test minus mean GFR_{NMR} in control, relative to mean GFR_{NMR} in control, across each pool). In case the mean relative bias was > ±10% or in case of missing valid measurements (red fields), an interference effect by the tested substance was considered, and a dose-response experiment was conducted to determine the concentration of spiked substance at which interference occurred (Figure 4). N/A, not applicable.
